# A Pulse Rate Detection Method for Mouse Application Based on Multi-PPG Sensors

**DOI:** 10.3390/s17071628

**Published:** 2017-07-14

**Authors:** Shu-Tyng Lin, Wei-Hao Chen, Yuan-Hsiang Lin

**Affiliations:** Department of Electronic and Computer Engineering, National Taiwan University of Science and Technology, Taipei 10607, Taiwan; stlinalice@gmail.com (S.-T.L.); m10302103@mail.ntust.edu.tw (W.-H.C.)

**Keywords:** pulse rate, PPG, multi-PPG sensor, computer mouse, vital sign monitoring

## Abstract

Heart rate is an important physiological parameter for healthcare. Among measurement methods, photoplethysmography (PPG) is an easy and convenient method for pulse rate detection. However, as the PPG signal faces the challenge of motion artifacts and is constrained by the position chosen, the purpose of this paper is to implement a comfortable and easy-to-use multi-PPG sensor module combined with a stable and accurate real-time pulse rate detection method on a computer mouse. A weighted average method for multi-PPG sensors is used to adjust the weight of each signal channel in order to raise the accuracy and stability of the detected signal, therefore reducing the disturbance of noise under the environment of moving effectively and efficiently. According to the experiment results, the proposed method can increase the usability and probability of PPG signal detection on palms.

## 1. Introduction

The statistic of World Health Organization (WHO) in 2016 has shown that the cardiovascular diseases (CVDs) are the number 1 cause of death globally [1]. A review of the European Society of Cardiology has mentioned that heart rate is an independent cardiovascular risk factor [2], while the American Heart Association (AHA) has noticed that to control the heart rate within a relatively normal range is one of the treatment goals [3]. Therefore, a comfortable and easy-to-use design of heart rate monitoring device will be the trend in the following years.

Photoplethysmography (PPG) is one of the optical techniques that has been developed for experimental use in vascular disease [4] and has applications on measuring the oxygen saturation, blood pressure, cardiac output [5], and determining heat stress level by using frequency analysis of PPG and its derivatives [6]. To compare PPG with other heart rate detection methods like electrocardiography (ECG), PPG is more convenient to use and easier to wear [5]. Users can easily wear the PPG devices on the earlobe, on the finger, on the wrist, or on the toe [7,8,9,10,11]. Different positions are chosen to detect the signal in order to improve the signal quality. Though PPG devices have advantages like being light-weighted, portable, and easy-to-use, motion artifacts are still the problems that have to overcome [5,12,13,14,15].

In the previous studies, problems such as weight of device, or whether the device is comfortable to wear or not are highly related to the acceptability and popularity of the users. Several later studies have proposed PPG detection on the side surface of the computer mouse [16,17,18]. However, users have to force their thumbs to contact the surface of the PPG sensor, which may not be comfortable. In this study, we suggest an alternate sensing position, developed by placing the multi-PPG sensor on the top surface of the mouse, which makes contact with the user’s palm. In this solution, multiple sensors can increase the contact area and the probability of signal detection, and users can use the mouse comfortably instead of forcing their finger skin to touch the sensor.

The PPG signal suffers from motion artifacts easily, and the signal quality is constrained by the position chosen for signal detection [10,19]. Lee et al. have indicated that the greatest and worst cause of artifact noise that contaminates the PPG signals is motion artifacts produced from physical activity and body movement [20]. In order to achieve clear PPG and improve the accuracy of pulse rate detection, filter and amplification are required [21].

Tran and Chung have provided a robust algorithm for peak detection that can eliminate the fake peaks and correct error peaks in the original PPG signal under challenging movement conditions in real time on a personal computer mouse [18]. Nevertheless, the error rate of the algorithm still increases due to mouse movements that cause more motion artifacts [14,15,20]. Alzahrani et al. and Warren et al. have constructed a design of multi-PPG sensors combining with an accelerometer on wearable devices to reduce motion artifacts [22,23]. These structures, however, have the disadvantages of increasing costs and algorithm complexities.

The purpose of our study is to implement a comfortable and easy-to-use module with a stable and accurate pulse rate detection on a computer mouse that most people are using everyday either in the office or at home, as multi-channel sensors can handle displacement and misalignment of the palm [22]. We proposed a multi-sensor module design (which is smaller than the previous mentioned studies [16,17,18]) on the top surface of the mouse, and other circuits including the microcontroller are inside the mouse. Therefore, users don’t have to force their hands holding the mouse while they are using the mouse. The multi-channel sensor structure can not only raise the area contacted by the users, but also increase the sensitivity and predictivity. Furthermore, a weighted average method for the mixed signal can emphasize the better signal with a higher weight and the poorer signal with a lower weight to improve the quality of the signal and to increase the accuracy of detection.

The paper is organized as follows: Section 2 describes the hardware structure and the proposed method to perform pulse rate detection based on a multi-sensor; the experimental device and the experimental procedures of each stage are stated in Section 3; Section 4 summarizes the experimental results of the proposed method and the comparisons to each channel and both the average and the weighted average method; and finally, some conclusions are discussed in Section 5.

## 2. Materials and Methods

### 2.1. System Architecture

The proposed structure consists of a microcontroller (NUC120, Nuvoton Technology Corporation, Hsinchu City, Taiwan, which is an ARM^®^Cortex™-M0-based microcontroller), a Bluetooth module, the four-PPG sensor with four sets of band-pass filter with an amplifier, and a programmable-gain amplifier (PGA117, Texas Instruments, Dallas, TX, United States). The signal is extracted from each PPG sensor (uPI Semiconductor, Hsinchu County, Taiwan, with a wavelength of 850 nm) as one signal channel, which includes an infrared LED and a photodiode (PD).

**Figure 1:**
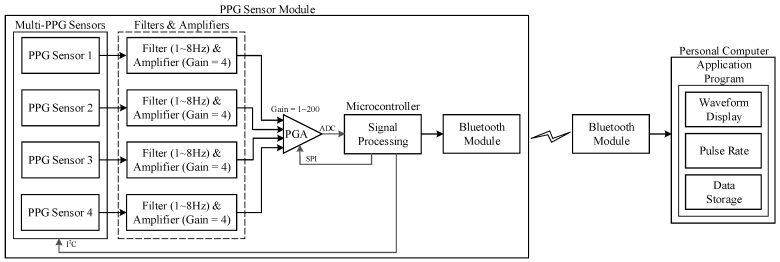
System structure.

The designed system structure is shown in Figure 1, which is based on the design of our previous study [24]. The LED emits infrared light to the skin, and then the photodiode receives the reflected signal that is dependent on the changes in the blood pulse and generates the PPG signal at the output of the sensor. The PPG signal will then input through a band-pass filter and an amplifier. The filtered and amplified signal will next input into a microcontroller to evaluate the pulse rate by using the algorithm and finally output the PPG waveform of each channel and a mixed PPG to a PC, which is a user interface, for waveforms and pulse rate display, and for health data storage.

### 2.2. Hardware Design

The Cortex™-M0 based microcontroller NUC120 (Nuvoton Technology Corporation, Hsinchu City, Taiwan) is used as the main controller in our design, and it is embedded in the computer mouse combined with other related circuits. The Inter Integrated Circuit (I^2^C) is used to control the PPG sensors, and the Serial Peripheral Interface (SPI) is used to control the PGA switching on modifying the magnitude factor of different PPG channels. The filtered and amplified PPG signal will input through a built-in analog-to-digital converter (ADC) with a resolution of 12 bits and the sampling rate is 200 Hz, and then the output signal will be communicated to the personal computer by the Bluetooth module. The waveform and the evaluated pulse rate will be displayed on the computer and the data is stored and can be analyzed by the application program.

**Figure 2:**
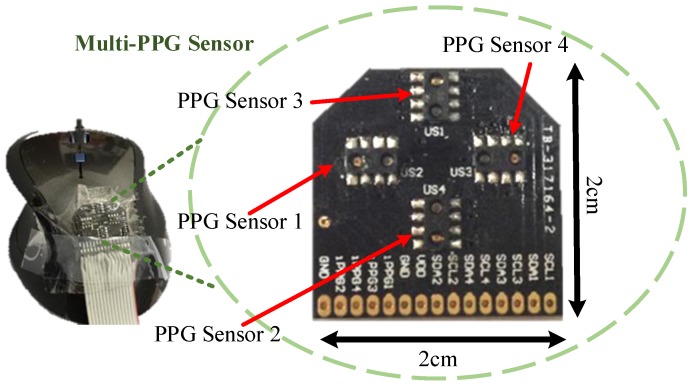
Views of the designed hardware device [24]: the external view of the proposed multi-PPG sensor with four channels placing on the top of the mouse.

In this work, we propose a pulse rate detection device using a computer mouse (TCN183, T.C. STAR, New Taipei City, Taiwan, with dpi of 800). Our proposed system is embedded in the internal of the mouse and only the multi-PPG sensor of the PPG sensor module is placed on the top surface of the mouse as shown in Figure 2 with 2 cm wide and 2 cm long. The PPG sensors 1, 2, 3, and 4 show the position of each PPG channel on the sensor, respectively.

### 2.3. Algorithm Development

**Figure 3:**
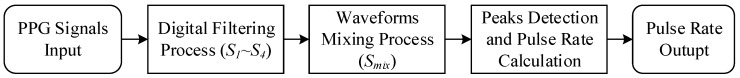
Signal processing flow in the microcontroller.

In the previous hardware structure section, we have acquired the PPG signals from the hardware. All algorithms including the peak detection, pulse rate calculation, and digital signal processing are done in the microcontroller. In order to improve the quality of the PPG acquisition, we first input the PPG signal into a digital filtering process to eliminate the noise. Then, a waveforms mixing process is to emphasize the needed PPG signal to increase the accuracy of peaks detection and pulse rate calculation. The system flow of the designed process in this paper is depicted in Figure 3.

#### 2.3.1. Digital Filtering Process

After analog signal processing in the previous hardware structure section, the extracted PPG signal has become more obvious and much cleaner than before, for which the signal is small and noise contaminated. However, there is still some high frequency noise disturbing the PPG signal due to hardware structure. Therefore, a 40th-order low-pass FIR (Finite Impulse Response) filter with a cutoff frequency of 5 Hz is designed to reduce the high frequency noise.

#### 2.3.2. Waveforms Mixing Process

Since blood vessels spreading in the palm are diverse, the difficulties of PPG detection may be increased according to the position of palm that contacts the PPG sensors. Therefore, this study proposes to expand the area of detection using the structure of multi-sensors, and then mix the signals of each channel to form a new PPG signal that improves the quality of the signal and makes the detection easier and more convenient.

Two methods are used as comparisons in our proposed design, the average, and the weighted average method, with the purpose of observing whether the weighted average method is a better way to improve signal quality than the average method.

- *Average Method:* In order to get the average of the waveforms, four channels of the signal processed PPG signals will be summed and then divided by the number of channels. The signal $S_{i}$ of each channel *i* by each sample point *n* are summed and then divided by the number of channels *i*, which is four in this case, and carried out the result of the calculated mixed signal $S_{mix}\left[n\right]$ as Equation (1):
  (1) $$S_{mix}\left[n\right]=\frac{\sum_{i=1}^{4}S_{i}\left[n\right]}{4}.$$
- *Weighted Average Method:* In this method, the peak detection (from Section 2.3.3) is applied in the beginning of each time instant and then the better quality signal channels will be emphasized by increasing their weights according to Equation (2):
  (2) $$S_{mix}\left[n\right]=\frac{\sum_{i=1}^{4}W_{i}\left[n\right]\times S_{i}\left[n\right]}{\sum_{i=1}^{4}W_{i}\left[n\right]}.$$
  The decision of adjustment is described as three cases:
  **Increasing Weight:** $W_{i}\left[n\right]=W_{i}\left[n-1\right]+\alpha$, the case of peak detected in the process of peak detection and the peak-to-peak interval (PPI) also satisfied the requested range. The decision weight of $W_{i}\left[n\right]$ and $W_{i}\left[n-1\right]$ represent the weights of the current and the previous sample point, respectively. Constant α affects the weight of each signal point and is set to 2. The adjusted weight $W_{i}\left[n\right]$ is constrained in the range of 2 to 20 in this study by empirical tests.
  **Decreasing Weight:** $W_{i}\left[n\right]=W_{i}\left[n-1\right]-\alpha$, if peak is detected, but the requested range of PPI is not satisfied, the peak will be recognized as a noise and the weight will be decreased; if no peak is detected in the requested range of PPI, a decreasing weight is also applied.
  **Unchanged Weight:** $W_{i}\left[n\right]=W_{i}\left[n-1\right]$, otherwise stated in the previous two cases.

Therefore, a mixed waveform of four channels can be decided by two different methods above. According to the result $S_{mix}\left[n\right]$ of each sample either in the average method Equation (1), or the weighted average method Equation (2), we can then combine all sample points from each method into a whole waveform $S_{mix}$.

#### 2.3.3. Peak Detection and Pulse Rate Calculation

**Figure 4:**
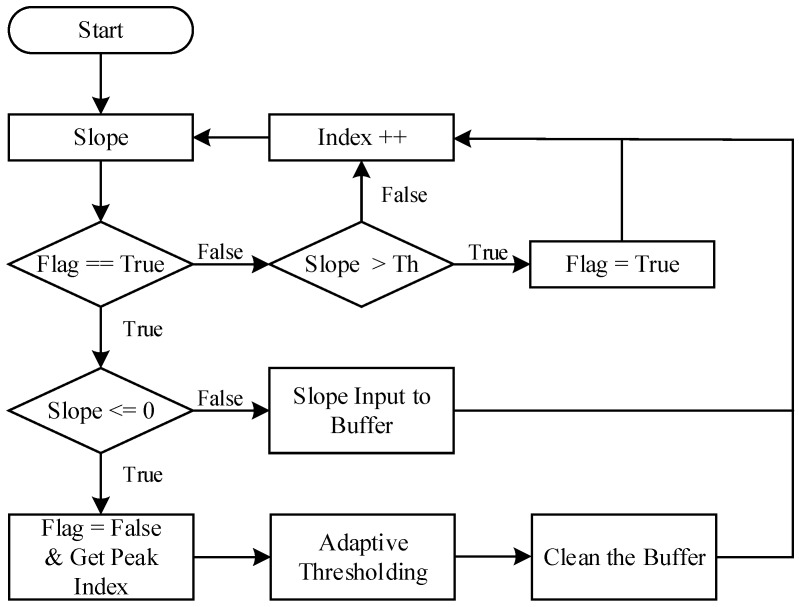
The peak detection flow, adapted from [25].

We can observe from the PPG signal that each pulse raises rapidly. Thus, the slope value of PPG signals and dynamic thresholding are used to detect the peaks. Figure 4 depicts the peak detection flow used in this study [25]. In the beginning of the test, the Flag is set to false. When the slope value is larger than the threshold (Th), the Flag is set to true and this slope is recorded in the buffer. While the Flag is true and the slope value is smaller than or equals to 0, a peak index (peak position) is recorded. The maximum slope value from the buffer will be used to update the new threshold as adaptive thresholding and then clean the buffer to restart the peak detection.

A PPI check is then applied after peak detection to prevent noise contaminations. When the PPI is in the requested range, a peak is detected and finally outputs the pulse rate (PR). The requested PPI range is set to PPI[n−1]×0.7<PPI[n]<PPI[n−1]×1.3 based on our previous work [25]. The pulse rate is calculated as Equation (3), where the PPI[n] is determined by the peak-to-peak interval between the *n*th and the (n−1)th peak and fs is the sampling rate. The peak detection and pulse rate calculation procedure are referenced and adapted based on our previous research [24,25]:

(3) $$PR\left[n\right]=\frac{60\times fs}{PPI[n]}.$$

## 3. Experimental Processes

### 3.1. Experiment Setup

A reference ECG lead I signal is measured by using electrodes on the right and the left wrists and are connected to the microcontroller to synchronized with the PPG signal [26,27]. In order to verify the performance of the proposed algorithm that forms the mixed signal, experiments are designed in four stages (six movements): rest, slow movement (horizontal and vertical), rapid movement (horizontal and vertical), and browsing stage; details will be described in the section of *Movement Stages*. Furthermore, the results of each channel and the mixed waveform of two methods: the average method and the weighted average method are shown as comparisons.

There are 21 healthy volunteered subjects who participated in the experiments. The demographic data of the volunteers in this study is listed in Table 1: 17 males and four females with the mean ± standard deviation age of 25.14 ± 5.66 years old (from 20 to 41), the weight is 70.19 ± 15.52 kg (from 45 to 101), the height is 170.24 ± 6.99 cm (from 155 to 183), and the average heart rate (HR) is 88.84 ± 10.63 bpm (from 66.74 to 102.60). Every subject has to perform four stages of experiments, in which six movements are included, with a recording time of one minute per movement. For the reason of the initialization of our proposed device and the reference device, the first and the last five seconds of recording time will be discarded before data analyzing.

### 3.2. Movement Stages

The main goal in this section is to verify whether the mixed signal of multiple channels from the average method and the weighted average method can have more stable signal than a single channel signal. In addition, the purpose of each stage is to test the accuracy of the proposed device across different experimental conditions—for instance, different speed and directions of movements.

#### 3.2.1. Rest Stage

Subjects should hold the mouse and make contact with their palms, without doing any actions like moving, or clicking, therefore controlling the variables.

#### 3.2.2. Slow Movement Stage

**Figure 5:**
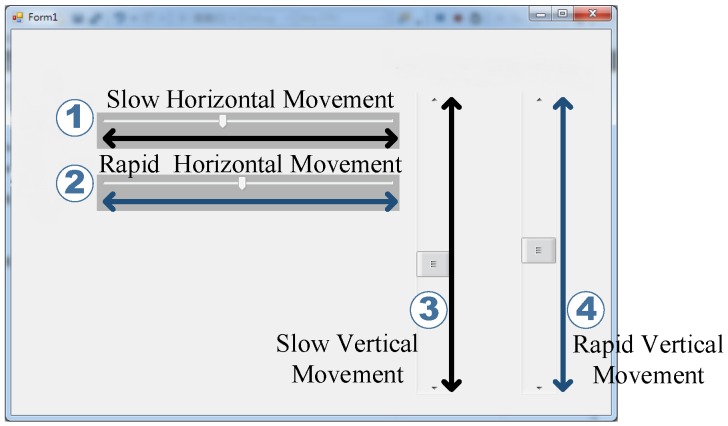
Experiment user interface: **1** is for slow horizontal movement, **2** is for rapid horizontal movement, **3** is for slow vertical movement, and **4** is for rapid vertical movement. The double arrow in the figure presents the direction of each relative movement. Adapted from [24].

Figure 5 shows the experimental user interface of the slow and rapid movement stages. The numbers 1 and 3 in the figure depict the slow horizontal (left and right) and vertical (up and down) movement, respectively. Furthermore, the numbers 2 and 4 in the figure depict the rapid horizontal and vertical movement, respectively. The scrollbar in each described movement (numbers 1 to 4) will move automatically with a constant speed (slow and rapid defined in Section 3.2.2 and Section 3.2.3, respectively) according to the direction shown by each bidirectional arrow.

In this experiment, subjects have to move the mouse cursor following the moving scrollbar shown on the numbers 1 and 3 in Figure 5, which is the experiment of the slow horizontal and vertical movement. The speed of slow movement experiments, either the horizontal or the vertical, is 10 s per round with a distance of 720 pixels (2 cm in the real movement) set by the authors. Furthermore, the recording time for each slow horizontal or vertical movement is one minute. The speed of both movements are recorded as well.

#### 3.2.3. Rapid Movement Stage

In this experiment, subjects have to move the mouse to follow the scrollbar shown on the numbers 2 and 4 in Figure 5, which is the experiment of the rapid horizontal (left and right) and vertical (up and down) movement. The speed of this experiment, either the horizontal or the vertical, is 2 s per round with a distance of 720 pixels, which is set by the authors. Furthermore, the recording time for each movement is one minute. The speed of both movements is recorded as previously.

#### 3.2.4. Browsing Stage

The subject has to use the computer mouse either with moving, or left clicking the mouse quickly to browse the electronic document with the speed of 10 s per page for one minute. With respect to data observation and analysis, the PPG raw data of the proposed device and the reference device will be recorded, and the clicking time and times of clicking will be documented simultaneously.

### 3.3. Results Verification

**Figure 6:**
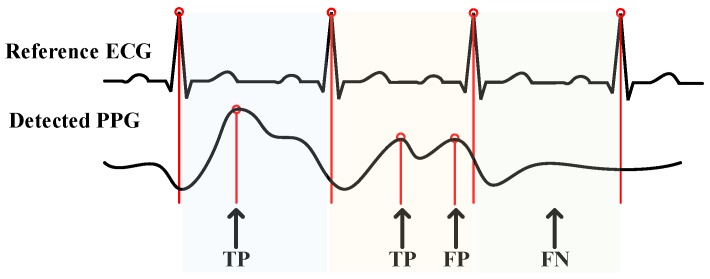
Definition of TP (True Positive), FP (False Positive), and FN (False Negative). Adapted from [24].

To evaluate the performance of the designed algorithm, the signal acquired from the proposed and the reference device have been compared and a sensitivity Se as Equation (4), positive predictivity +P as Equation (5), and failed detection rate FDR as Equation (6) test has been carried out for different configuration parameters [18]:

(4) $$\begin{array}{cc} Se= & \frac{TP}{TP+FN}\times 100\%, \end{array}$$

(5) $$\begin{array}{cc} +P= & \frac{TP}{TP+FP}\times 100\%, \end{array}$$

(6) $$\begin{array}{cc} FDR= & \frac{FP}{TP}\times 100\%, \end{array}$$

where TP (True Positive) is the number of peaks detected; FN (False Negative) is the number of peaks non-detected; and FP (False Positive) is the number of artifacts or noise that classified as peaks. The definition of each parameter is shown as Figure 6.

Different parameter presents different meaning: the higher the Se, the lower probability of the peaks non-detected; +P represents the ratio of true peaks detected, which means the correctness of the detection; and the FDR is the ratio of failure to correction. Otherwise, if high Se and low FDR are evaluated, the detection result is contributed to a high accuracy.

## 4. Results and Discussion

### 4.1. Accuracy Evaluation and Comparison

In order to provide a comparison of the proposed algorithm, we compare the acquired PPG signal from our proposed device and the reference ECG signal from the reference device to evaluate the configuration parameters Se, +P, and FDR.

Table 2 summarizes the results of the Se, +P, and FDR of each channel and the mixed signal under the rest environment for all subjects. The performance of the mixed signal from the average method has an Se of 98.50% and an FDR of 0.15%, and the performance of the weighted average method has an Se of 99.71% and an FDR of 0.00%. While the worst performance under the rest environment is the signal from Channel4, it still has the Se of 93.05% and a low FDR of 1.77%. Therefore, a high Se and a low FDR of the proposed system is proved under the rest environment.

In the stages of slow movement and rapid movement, we have performed two directions of each stage: the horizontal and the vertical movement. Table 3 and Table 4 show a comparison among the results of the FDR (%) of each channel and the mixed waveform from the average method and the proposed method of every subject for the slow horizontal movement and the rapid horizontal movement, respectively. The average FDR of each channel and both mixed waveform methods of all subjects are also listed.

**Figure 7:**
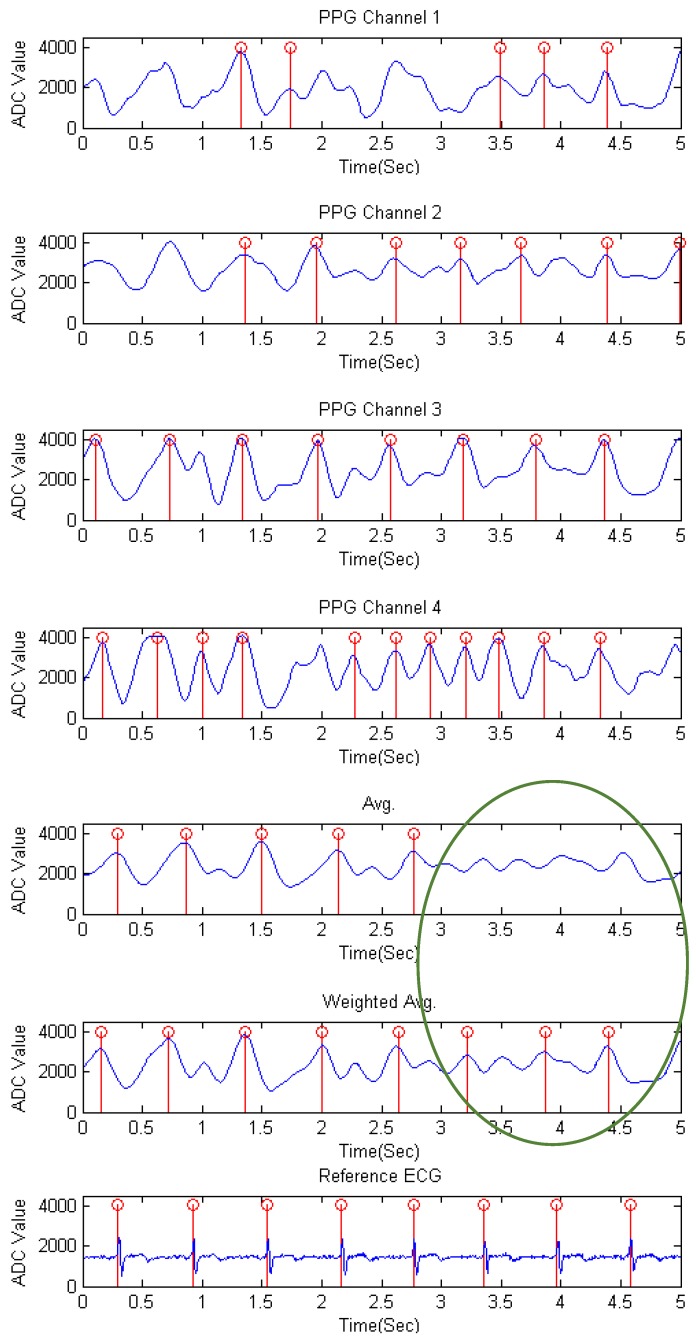
The waveforms of the rapid movement stage of each channel, the mixed signals by the average method and the weighted average method, and the reference ECG signal for subject 6.

The waveforms of the rapid movement stage of each channel and the mixed signal by the average and the proposed weighted average method for subject 6 is shown in Figure 7. As stated in the previous research [26], a PPG peak comes after the ECG R-wave in the same cardiac cycle. We can obviously observe from each channel that there may be more than one peak appearing between the ECG R-peak and the following R-peak, which may affect the accuracy of detection. Moreover, the signal channels are motion contaminated due to a rapid movement of the mouse, and the peak detection result of the mixed signal by the average method is still affected by the fluctuations of motions, while the proposed weighted average method is almost close to the reference ECG signal. In the circle where some non-detected peaks of the average method occurred is due to the fact that the slope between the time period of 3 to 3.5 s does not exceed the maximum slope and leads to undetected peaks in the following few samples, while the weighted average method does amplify those points and detected the peaks.

**Figure 8:**
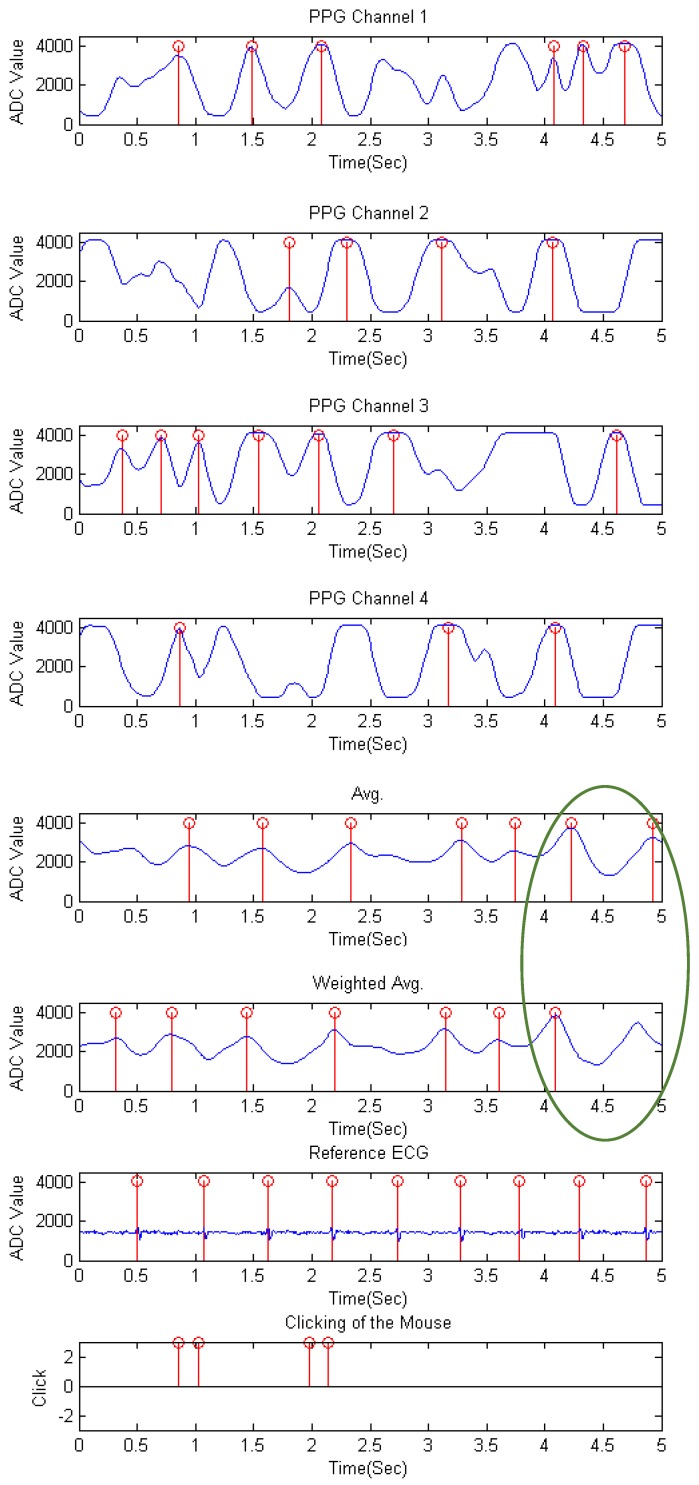
The waveforms of the browsing stage of each channel, the mixed waveforms by the average and the weighted average method, the reference ECG signal, and the clicking of the mouse for subject 9.

Table 5 is the FDR (%) result of each channel and the mixed waveform from the average and the weighted average method of every subject for the browsing experiment. The combination results of each channel and both mixed waveform methods of all subjects are also listed. As can be observed, though the low FDR channels differ from subjects, the evaluated performance of the proposed weighted average method still results in lower failure of detection than the mixed signal of the average method. The waveform of the browsing stage for subject 9 is shown as Figure 8. It is shown that the signal is disturbed by moving and clicking. Especially in Channel3, the consecutive fluctuations in 0.5 to 2 s appear when clicking occurs. In the circle is the period where current PPI exceeds the requested range and therefore comes out as a non-detected peak in the weighted average method.

Table 6 and Table 7 summarized the results of all experiment stages by the average method and the weighted average method of all subjects, respectively. Comparing the FDR of each method, we can observe that the proposed weighted average method has lower FDR than the average method. The proposed method also has higher Se and +P than the average method of all experiment stages, as it was known that a higher Se is desirable to provide better prediction accuracy [28]. The +P is used to assess the repeatability, the precision, and the reproducibility of the methods, and a higher +P is desirable to provide better performance [29]. Therefore, it can be concluded from the results that a mixed waveform by the weighted average method has better performance including high accuracy and detection rate on peak detection than the average method.

### 4.2. Effect of Sensor Positioning

In the four stages of six movements, the better performance signal channel differs from each subject. However, after the process of waveform mixing by the proposed algorithm, the Se and the FDR of the mixed signal maintain the best or the second best. These have proved that even different habits of mouse use will affect the position detected, and the structure of multi-sensor can increase the probability of signal extraction.

### 4.3. Effect of Moving and Clicking of the Computer Mouse

There are two important movements of mouse usage: moving and clicking. From the stage of slow movement and rapid movement in the previous section, more motion artifacts are found in rapid movement stage than in slow movement stage. Though the sensitivity and the detection rate are reduced for rapid movement stage either for the results of the proposed algorithm or the average method, the evaluated result still remains a high Se and a low FDR.

In the browsing stage, the tester has to use the mouse to browse the electronic document that combines mouse clicking and moving, therefore causing the decreasing of the sensitivity as compared to the rest environment. The reason for the loss of sensitivity is that the palm may leave a distance from the sensor when clicking occurs; thus, unexpected peaks appear and the difficulties of detection also increase. From the result of browsing by the proposed method in Table 5, failure of detection appears in most channels for almost all the subjects, but this issue is overcome with the proposed method, which will amplify the cleaner signal and reduce the effects of noise. The overall results of the proposed method in Table 7 also show the Se and the FDR of the detected PPG signal.

### 4.4. Eye Safety Concern and Power Saving

As optical measurement like PPG has been widely used nowadays, a growing concern about the possible use of LED arrays that might pose a potential threat has been derived in some studies [30]. Concerning the eye safety related to the power of infrared LEDs when the palm is not covering the mouse, a power saving mode is designed in this study to detect whether the user’s palm is contacting the mouse or not. When no palm (pulse rate) is detected, the turn-on time of the LED lights will be reduced by lowering its modulation frequency. Therefore, the controls of the PPG sensors are added not only for power saving, but also for protecting our eyes.

### 4.5. Limitations of the Study and Future Works

There are still some limitations in this study. The limitation of the contact sensor is that the sensors must be contacted with the palm while doing the measurement. In the normal computer use, the signals recorded with the system can be relatively piecewise periods of PPG signals since the user often loosens his/her grip on the mouse when he/she is typing the text with a keyboard. Therefore, the PPG signals were recorded only when the user is gripping the mouse.

The results in the study are carried out by limits of young and healthy subjects; thus, in the future, more subjects from different ages (young to old) and more conditions, like small and large palms, people with poorer circulation, or people with arrhythmias will be tested.

Moreover, motion artifacts still negatively impact measurement accuracy. Therefore, how to determine the signal quality is important [12,31,32]. In the results of the experiments, we found some sensitivity loss in the movement stages, which is due to the peaks being non-detected. Therefore, a more robust peak detection by event-related moving averages with dynamic threshold is considered [13] to improve the performance of peak detection in the future.

## 5. Conclusions

A real-time pulse rate monitoring mouse with a multi-sensor structure has been proposed in this study, and a weighted average method to adjust the weight of the signal according to the quality of the channel, which increase the accuracy and the stability of PPG peak detection. The mixed waveform process by the proposed method emphasizes the cleaner signal, therefore reducing the disturbance of noise effectively.

Despite the performance results by the weighted average method of each subject possibly not being the best, the overall sensitivity and the failed detection rate of the processed signal outperform most single channels. The four experiment stages have proved that the proposed multi-sensor structure and the weighted average method for the mixed waveform process can raise the usability of the detected PPG signal efficiently and effectively, therefore increasing the probability of PPG signal detection on palms.

Since the proposed design is simple, easy-to-use, and low-cost, the device could be served as a measurement tool to collect physiological signals and play a major role in early detection of diseases. Therefore, the proposed design is suitable as a solution to overcome barriers to improve health care in low- and middle-income countries [33].

## Figures and Tables

**Table 1:** Demographic data for the participants in this study.

Characteristic	Mean	SD	Min	Max
Age (yrs)	25.14	5.66	20	41
Weight (kg)	70.19	15.52	45	101
Height (cm)	170.24	6.99	155	183
BMI (kg/m^2^)	24.05	4.49	18.43	35.36
Resting HR (bpm)	88.84	10.63	66.74	102.60

**Table 2:** Comparisons of the results of sensitivity, positive predictivity, and failed detection rate for the mixed signal for both the average method (Avg.), weighted average method (WeightedAvg.), and each channel under the rest environment.

Signal	Se(%)	+P(%)	FDR(%)
Channel1	95.61	99.18	0.83
Channel2	96.85	99.85	0.15
Channel3	99.71	100.00	0.00
Channel4	93.05	98.26	1.77
Avg.	98.50	99.85	0.15
WeightedAvg.	99.71	100.00	0.00

**Table 3:** Failed detection rate (%) of each subject for slow horizontal movement.

**Signal**	**Sub1.**	**Sub2.**	**Sub3.**	**Sub4.**	**Sub5.**	**Sub6.**	**Sub7.**	**Sub8.**	**Sub9.**	**Sub10.**	**Sub11.**
Channel1	1.92	4.65	4.55	3.08	1.39	0.00	0.00	0.00	0.00	1.79	0.00
Channel2	0.00	4.00	22.73	1.52	0.00	6.56	0.00	0.00	9.09	0.00	20.83
Channel3	0.00	0.00	0.00	6.67	0.00	2.82	0.00	0.00	0.00	0.00	0.00
Channel4	0.00	11.11	10.64	7.02	2.67	4.23	0.00	0.00	24.32	5.77	7.81
Avg.	0.00	3.70	11.11	3.17	1.18	2.70	0.00	0.00	0.00	7.27	1.54
WeightedAvg.	0.00	0.00	0.00	1.64	0.00	0.00	0.00	0.00	0.00	0.00	0.00
**Signal**	**Sub12.**	**Sub13.**	**Sub14.**	**Sub15.**	**Sub16.**	**Sub17.**	**Sub18.**	**Sub19.**	**Sub20.**	**Sub21.**	**All**
Channel1	0.00	11.32	3.39	1.82	0.00	3.33	9.43	0.00	0.00	0.00	1.95
Channel2	1.92	0.00	7.84	1.69	1.04	0.00	9.09	0.00	5.77	0.00	3.65
Channel3	0.00	0.00	0.00	1.64	1.09	0.00	0.00	0.00	0.00	3.33	0.74
Channel4	11.76	1.67	32.14	4.08	1.03	5.36	7.84	1.16	2.17	0.00	5.93
Avg.	0.00	0.00	1.69	5.56	0.00	1.56	0.00	0.00	0.00	1.61	1.58
WeightedAvg.	0.00	0.00	0.00	0.00	0.00	1.54	0.00	0.00	0.00	0.00	0.15

**Table 4:** Failed detection rate (%) of each subject for rapid horizontal movement.

**Signal**	**Sub1.**	**Sub2.**	**Sub3.**	**Sub4.**	**Sub5.**	**Sub6.**	**Sub7.**	**Sub8.**	**Sub9.**	**Sub10.**	**Sub11.**
Channel1	0.00	0.00	2.50	7.69	4.17	24.32	7.14	0.00	0.00	0.00	1.72
Channel2	0.00	0.00	6.06	1.64	0.00	8.16	0.00	1.85	2.00	0.00	15.56
Channel3	9.43	4.08	0.00	0.00	5.36	3.57	0.00	0.00	1.61	0.00	0.00
Channel4	1.89	0.00	12.20	1.92	1.69	20.00	4.55	4.26	8.33	8.62	7.69
Avg.	0.00	6.67	9.09	0.00	2.04	15.22	7.14	0.00	6.67	0.00	1.79
WeightedAvg.	0.00	1.89	8.89	0.00	0.00	2.00	0.00	0.00	1.85	0.00	0.00
**Signal**	**Sub12.**	**Sub13.**	**Sub14.**	**Sub15.**	**Sub16.**	**Sub17.**	**Sub18.**	**Sub19.**	**Sub20.**	**Sub21.**	**All**
Channel1	0.00	1.67	1.49	2.13	0.00	0.00	13.64	0.00	6.98	0.00	2.73
Channel2	0.00	0.00	6.90	0.00	0.00	3.45	0.00	0.00	6.67	3.57	2.40
Channel3	0.00	0.00	0.00	4.35	1.41	0.00	11.11	0.00	1.96	0.00	1.82
Channel4	6.25	0.00	4.69	9.30	2.27	5.08	4.44	0.00	3.39	0.00	4.75
Avg.	0.00	0.00	5.36	11.11	2.90	0.00	3.92	0.00	5.13	1.43	3.20
WeightedAvg.	0.00	0.00	1.72	0.00	1.30	0.00	0.00	0.00	2.13	0.00	0.83

**Table 5:** Failed detection rate (%) of each subject for browsing.

**Signal**	**Sub1.**	**Sub2.**	**Sub3.**	**Sub4.**	**Sub5.**	**Sub6.**	**Sub7.**	**Sub8.**	**Sub9.**	**Sub10.**	**Sub11.**
Channel1	1.79	0.00	4.88	0.00	1.41	2.67	0.00	11.90	2.27	0.00	0.00
Channel2	3.28	1.61	7.32	1.41	0.00	3.95	0.00	1.96	3.57	1.54	6.56
Channel3	0.00	1.59	3.57	5.26	0.00	2.90	0.00	0.00	4.44	0.00	0.00
Channel4	0.00	3.45	15.22	1.45	1.45	5.56	0.00	6.12	2.08	4.62	22.92
Avg.	3.45	0.00	31.58	0.00	0.00	2.67	0.00	0.00	8.93	0.00	0.00
WeightedAvg.	0.00	0.00	0.00	0.00	0.00	2.56	0.00	0.00	0.00	0.00	0.00
**Signal**	**Sub12.**	**Sub13.**	**Sub14.**	**Sub15.**	**Sub16.**	**Sub17.**	**Sub18.**	**Sub19.**	**Sub20.**	**Sub21.**	**All**
Channel1	0.00	3.28	9.38	2.30	1.47	7.81	0.00	0.00	5.00	0.00	2.08
Channel2	1.72	0.00	1.59	1.22	7.27	10.17	0.00	0.00	16.00	0.00	3.01
Channel3	0.00	0.00	0.00	2.63	11.29	12.90	1.79	3.39	1.72	0.00	2.44
Channel4	8.33	0.00	4.92	5.41	1.35	5.36	7.55	1.43	16.33	0.00	5.03
Avg.	0.00	0.00	1.47	2.63	1.59	3.85	0.00	0.00	3.28	0.00	2.18
WeightedAvg.	0.00	0.00	0.00	1.28	1.47	2.00	0.00	0.00	2.00	0.00	0.44

**Table 6:** Results of sensitivity, positive predictivity, and failed detection rate of all experiment stages of all subjects by the average method.

Movements	Se(%)	+P(%)	FDR(%)
Rest	98.50	99.85	0.15
Slow horizontal movement	92.37	98.45	1.58
Slow vertical movement	81.58	95.85	4.33
Rapid horizontal movement	81.42	96.90	3.20
Rapid vertical movement	77.48	97.36	2.71
Browsing	87.58	97.87	2.18

**Table 7:** Results of sensitivity, positive predictivity, and failed detection rate of all experiment stages of all subjects by the weighted average method.

Movements	Se(%)	+P(%)	FDR(%)
Rest	99.71	100.00	0.00
Slow horizontal movement	94.24	99.85	0.15
Slow vertical movement	87.34	98.83	1.19
Rapid horizontal movement	84.26	99.17	0.83
Rapid vertical movement	84.36	98.12	1.91
Browsing	89.04	99.56	0.44

## Abbreviations

The following abbreviations are used in this manuscript:

PPG	Photoplethysmography
WHO	World Health Organization
CVD	Cardiovascular Disease
AHA	American Heart Association
ECG	Electrocardiography
PD	Photodiode
I^2^C	Inter Intergrated Circuit
SPI	Serial Peripheral Interface
ADC	Analog-to-Digital Converter
FIR	Finite Impulse Response
PPI	Peak-to-Peak Interval
PR	Pulse Rate
BMI	Body Mass Index
Se	Sensitivity
+P	Positive Predictivity
FDR	Failed Detection Rate
TP	True Positive
FN	False Negative
FP	False Positive
